# Construction and validity of an educational video on rapid HIV testing for young black people

**DOI:** 10.1590/0034-7167-2024-0409

**Published:** 2025-10-03

**Authors:** Nikaelly Pinheiro Mota, Marli Teresinha Gimeniz Galvão, Nuno Damácio de Carvalho Félix, Renata Karina Reis, Ivana Cristina Vieira de Lima Maia, Rosely Leyliane dos Santos, Juliana Cunha Maia, Jéssica Karen de Oliveira Maia

**Affiliations:** I Universidade Federal do Ceará. Fortaleza, Ceará, Brazil; II Universidade Federal do Recôncavo da Bahia. Santo Antônio de Jesus, Bahia, Brazil; III Universidade de São Paulo. Ribeirão Preto, São Paulo, Brazil; IV Universidade Estadual do Ceará. Fortaleza, Ceará, Brazil; V Universidade Regional do Cariri. Crato, Ceará, Brazil

**Keywords:** Instructional Film and Video, HIV Testing, Validation Study, Black People, Nursing, Película y Video Educativos, Prueba de VIH, Estudio de Validación, Población Negra, Enfermería

## Abstract

**Objectives:**

to develop and validate an educational video on rapid HIV testing for young black people.

**Methods:**

technological production research, developed through the following stages: 1) literature review; 2) script development; 3) content validity; 4) video development; and 5) appearance validity. The Content Validity Index (CVI), Concordance Index (CI) and exact binomial test were calculated, considering p>0.05 and a proportion of 0.80 of agreement.

**Results:**

the video was three minutes and 22 seconds long and covered rapid HIV testing with a visual design that included animation, subtitles, and narration in Brazilian Sign Language (LIBRAS). The overall CVI of the video was 0.90 (p-value=0.38; 95% CI: 0.46-0.90), confirming its suitability and validity.

**Conclusions:**

the educational technology, in the form of a video, on rapid HIV testing for young black people is valid in terms of content and appearance, with potential usefulness for health education actions.

## RESUMEN

**Objetivos:**

crear y validar un vídeo educativo sobre pruebas rápidas de VIH para jóvenes negros.

**Métodos:**

investigación de producción tecnológica, desarrollada a través de las siguientes etapas: 1) revisión de literatura; 2) construcción del guión; 3) validación de contenido; 4) construcción de video; y 5) validación de la apariencia. Se calculó el Índice de Validez de Contenido (IVC), Índice de Concordancia (IC) y prueba binomial exacta, considerando p>0,05 y la proporción de concordancia de 0,80.

**Resultados:**

el vídeo tiene una duración de tres minutos y 22 segundos, abarca la prueba rápida del VIH y el diseño visual incluye animación, subtítulos y narración en Lengua de Señas Brasileña (LIBRAS). El CVI total del vídeo fue de 0,90 (p-valor=0,38; IC 95%: 0,46-0,90), lo que confirma su idoneidad y validez.

**Conclusiones:**

la tecnología educativa, en formato de vídeo, sobre la prueba rápida del VIH para jóvenes negros, es válida en términos de contenido y apariencia, con potencial utilidad para acciones de educación en salud.

## INTRODUCTION

The social vulnerability experienced by black people causes health disadvantages, due to some factors such as low educational and socioeconomic levels, the number of people under financial dependency and difficulty in accessing healthcare services being more prevalent in these individuals, increasing the risk of exposure to HIV infection^(1)^.

Epidemiological evidence indicates that, between 2007 and 2021, the HIV infection rate was higher among black people, being 51.7% among black and mixed racepeople^(2)^.Therefore, there is a higher percentage of HIV infection cases among young people, who become more vulnerable to infection by the virus due to various situations, such as early sexual initiation, desire for acceptance and inclusion in social groups, use of alcohol and other drugs, gender issues, in addition to less concern about acquiring sexually transmitted infections (STIs)^(3)^.

That said, the relevance of nursing care is highlighted as it occupies a strategic position to favor the HIV epidemic control among the young and black population, recognizing and understanding the social representations of this population regarding vulnerabilities and risk factors inherent to infection^(4)^.

In order to allow greater dissemination of information about rapid HIV testing for young black people, we highlighted the use of educational videos that, through audiovisual elements such as sound, visual and textual stimuli, make technology favorable for the health promotion process. Moreover, video is an easy-to-use technology, encouraging curiosity, enabling participant interaction and allowing them to fast-forward, repeat and pause according to learning demands and pace^(5)^.

Scientific evidence mapped educational technologies developed for the black population, identifying technologies such as courses, messages, dramatization, applications, pamphlets, media campaigns, radio campaigns, Facebook^®^ groups and videos as interventions with the black population, with positive impacts^(6)^, but with a gap in knowledge about the creation of valid educational videos for the black population involving HIV testing. The novelty and originality of this study are highlighted, given that no national research published for the black public was found regarding the development of educational technology that promotes knowledge about rapid HIV testing.

In this sense, the scientific and social relevance of this research is based on the need for public health policies that aim to provide the expansion of care strategies, such as the use of educational videos and appropriate interventions to combat HIV in this population. Therefore, nursing care, combined with and reoriented to these strategies, becomes extremely relevant for the dissemination of practices aimed at the construction of health promotion and prevention.

## OBJECTIVES

To create and validate an educational video on rapid HIV testing for young black people.

## METHODS

### Ethical aspects

The research received a favorable opinion from the *Universidade Federal do Ceará* Research Ethics Committee, as established by Resolution 466/12^(7)^.

### Study design and period

This is applied research into technological production^(8)^, which involved the construction and validity of an educational video on rapid HIV testing for the young black population.

The stages taken in the study involved: 1) Scoping review to survey the literary *corpus* ^(6)^; 2) Storyline and script construction for the educational video; 3) Video script content validity with health judges and the young black population (target audiencerepresentatives); 4) Educational video construction; and 5) Educational video appearance and technical characteristic validity with health and social communication judges. The research followed the SQUIRE 2.0 (Equator Network) guidelines to guide the methodology, and was carried out from December 2021 to October 2023.

### Population or sample; inclusion and exclusion criteria

To develop the video, it was necessary to involve a specialized multidisciplinary team, which consisted of: the author of the research for developing the storyline; a scriptwriter, responsible for writing the script; a content reviewer, in charge of reviewing the script and videos; an audio and video producer, in charge of producing the video with subtitles and voiceovers for the characters; and an interpreter, to translate the video into Brazilian Sign Language (LIBRAS).

The validity process took place in two stages: 1) In script validity (with health judges who are experts in the subject and target audiencerepresentatives, who were young black people aged 18 to 29); and 2) In videovalidity (with health and social communication judges).

At both validity times, the process occurred through non-probabilistic, intentional, snowball sampling^(8)^. Judge selection was carried out by analyzing the resumes available in the Brazilian National Council for Scientific Development (In Portuguese, *Conselho Nacional de Desenvolvimento Científico* -CNPq) database and their professional experience in the respective areas. Inclusion criteria for judges were people with clinical experience and/or research developed in the area in question, experts in the conceptual structure involved and methodological knowledge about the technology to be validated. Concerning the number of judges required for the validity process, there is no established consensus in literature^(9)^.

Scoring criteria for selecting health judges considered the area of interest related to HIV/AIDS/STIs and rapid testing: having a doctoral degree in an area related to the topic of interest (4 points); having a thesis in the area of interest (2 points); having a master’s degree in an area related to the topic of interest (3 points); having a dissertation in the area of interest (2 points); having an article published in a journal on the area of interest (1 point); having professional practice of at least one year in the area of interest (2 points). A minimum score of five points was required to be invited to participate in the study^(10)^.

In script validity, 25 health judges were invited, but only 13 agreed to participate in the study, and 15 young black people, obtaining ten confirmatory responses. In video validity, the same 25 health judgeswere invited, but only 12 continued with the assessments, and as for social communication judges, 27 were invited, but only seven participated in the study.

### Study protocol

To create the video, recommendations from the methodological framework for developing audiovisual technologies were followed, occurring in three phases: pre-production (scoping review, storyline creation, script development and script validity); production; and post-production^(11)^.

During pre-production, a scoping review was developed with the aim of surveying the literary *corpus* on the main technologies developed for the black population in the context of HIV, in order to collaborate with the scientific basis of the video’s thematic subjects. The review sought to answer the following question: what are the educational technologies implemented for HIV prevention in black people?

This review process took place between December 2021 and January 2022, with a broad search being carried out through the Coordination for the Improvement of Higher Education Personnel (In Portuguese, *Coordenação de Aperfeiçoamento de Pessoal de Nível Superior* - CAPES) journal portal, accessing the MEDLINE/PubMed, Embase, LILACS, CINAHL, Scopus and Cochrane databases^(6)^.

The storyline content was developed by the researchauthor, and the script was subsequently created. The script was prepared by selecting guidelines made available by the Ministry of Health regarding the Combined Prevention Mandala, specifically on the item designated for rapid testing. Associated with the Ministry of Health guidelines, the script content was also based on results obtained through the review.

The storyline was constructed using general information about what was essential to find in the video. There is no guide in literature to guide this construction stage; therefore, its development was at the researcher’s discretion.

The pre-production phase continued with content validity by selected judges. The adapted questionnaires used for content validity were characterized by being Likert scale instruments, with items or statements to be assessed by professionals and the target audience^(12)^.

The questionnaires were inserted into Google Forms^®^, associated with the following documents: invitation letter, Informed Consent Form (ICF) and Confidentiality Agreement. The ICF was obtained from all judges involved in the study online, and it was accessed via a link to request acceptance to participate so that judges would receive a copy of the ICF in their email. A period of 15 days was granted for the return of the data collection material. After this period, the research author carried out data collection.

In total, two questionnaires were used to validate the script, one specifically for health judges, which assessed the technology objective, organization and relevance, and another for the target audience, which sought to assess the writing objectives, organization, and comprehension and style. After the analysis by experts/target audience, suggestions, corrections and/or recommendations that they considered important were added.

In the production stage, the educational video was developed through an audio and video production, recording and editing studio. The video was created using cartoons, presenting the inclusion of LIBRAS.

After the video was created, the validity process was repeated with the same health judges, and there was also the participation of technicianjudges(social communication professionals with expertise in video production). As with script validity, a 15-day period was granted to return the data collection material, with the possibility of adding suggestions, corrections and/or recommendations that were pertinent.

The post-production phase sought to share the video on the main digital platforms, such as YouTube^®^ (https://www.youtube.com/watch?v=-2RK6ufFNLI), WhatsApp^®^ and Instagram^®^.

### Analysis of results, and statistics

Analysis was performed using data from each evaluator, both from expert judges (health and social communication) and from the target audience. Descriptive statistics were used to analyze the characteristics of responding judges and responses to the script and video assessment questionnaires.

The Content Validity Index (CVI) was calculated for each item of the script/video assessment instrument, the domains of script/video assessment instrument and the global CVI (instrument as a whole). The Concordance Index (CI) was calculated, considering items that obtained a minimum agreement level of 75% in positive responses as validated^(8)^. To calculate the CI, the parameter used was the number of times the evaluators agreed on each of the answers, divided by the total number of assessments, whose result varied between 0% and 100%^(13)^.

The item whose agreement among judges was ≥0.80 was considered valid^(8)^.To verify the agreement among judges, the binomial test was used, considering that there was agreement among judges when p>0.05. The analyses were performed using the Jamovi version 2.3.

## RESULTS

**Figure 1:**
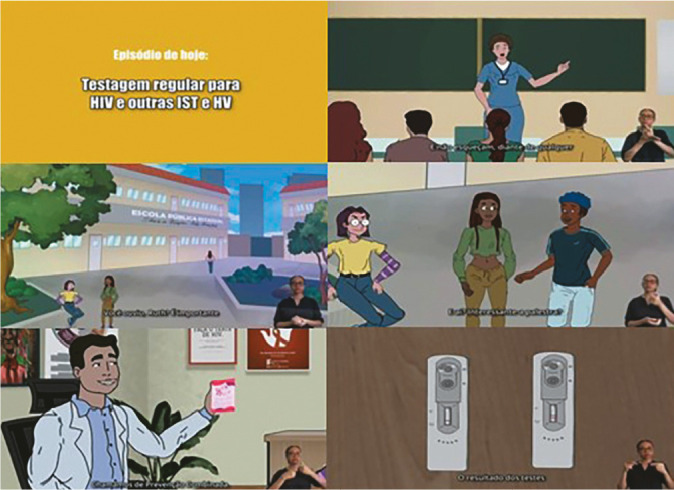
Scenes from the video about rapid HIV testing for young black people, Fortaleza, Ceará, Brazil, 2024

The video was three minutes and 22 seconds long and was titled “Regular testing for HIV and other STIs and HV”. It is available on some social media (Instagram^®^, WhatsApp^®^, Facebook^®^ and YouTube^®^). The video components (animation, subtitles and narration in audio and in libras) were synchronized, and an interpreter was displayed in the lower right corner, as shown in Figure 1.

The validity process took place in two stages: script validity by health judges and the target audience; and the second validity took place after the video was developed by the same health judges and by social communication judges with expertise in video production.

Thirteen judges participated in script validity by health judges, the majority of whom were female (69.2%), had nursing as their profession (92.3%) and worked as a nursing assistant at the institution (69.2%). As for qualifications, more than a third reported having a master’s degree (46.1%).

The total CVI of the assessment instrument was 0.8; however, p-value was equal to p=0.02, indicating that there was no agreement among all judges regarding the video scriptsuitability.

One of the judges stated that the script was adequate in terms of encouraging young people to be tested. However, he suggested that the counseling section could include information about using condoms during all sexual intercourse (oral, vaginal or anal) and about other forms of HIV infection. Another suggestion was that it could include information about ways to acquire the virus and how to prevent it (internal or external condoms during all sexual intercourse - oral, vaginal or anal). Another judge emphasized that it is important to improve the context of the scenes, including specific aspects of the target audience.

It should be noted that the video in this study aims to address “rapid testing”, not the use of condoms. Thus, some notes were of utmost importance for necessary changes and adjustments in the second version of the script, such as adding more specificity in the scenes regarding the target audience’s ethnic and racial characteristics.

The second script validity through the target audience (young black people). As for target audience characterization (n=10) that participated in the scriptvalidity, the predominant gender was female (70%), mixed race (70%), with complete (40%) or incomplete higher education (40%), with 40% being nursing technicians and 30% reporting nursing as their profession. There was unanimous agreement (100%) by the target audience regarding the script. The total CVI of responses to the video assessment instrument by the target audience was 0.9 (p=0.4), demonstrating the target audience’s agreement regarding the video scriptadequacy.

In relation to educational videovalidity, the results of CVI, CI and binomial test in judge validity were assessed in the following categories: content; audiovisual; and characters.

Health judges were predominantly female (75%), with half (50%) of mixed race/color and half of white race/color (50%). Most of respondents said that they had nursing as a profession (83.3%) and worked as a clinical nurse (66.7%). Regarding qualifications, 41.7% had a master’s degree, and 41.7% had a doctoral degree. None of the participants reported having previously published articles on the black population’s health.

Table 1 shows the CVI, CI and binomial test in relation to video validityby health judges. According to the observations, all individual items of the video assessment had a CVI greater than 0.8 and a CI greater than 80%. This was confirmed when performing the binomial test, which demonstrated agreement among health judges in relation to all items of the video assessment instrument, with all values greater than 0.05. The total CVI of the responses to the video assessment instrument was 0.8 (p=0.06), demonstrating health judges’agreement regarding video adequacy.

**Table 1:** Content Validity Index, Concordance Index and binomial test in relation to video validity by health judges (n=12), Fortaleza, Ceará, Brazil, 2024

Instrument questions	CVI	CI (%)	*p* value^*^	95% Confidence Interval
**Objective**	0.9537	95.37	0.0856	0.2120-0.8630
1.1 The information/content is coherent.	1.0000	100.00	0.1413	0.7353-1.0000
1.2 The information/content is presented in a clear and understandable manner.	0.9167	91.67	0.4803	0.6152-0.9978
1.3 The way the content is presented in the video is inviting to the viewer.	0.9167	91.67	0.4803	0.6152-0.9978
1.4 It can be circulated in the scientific community of the area.	1.0000	100	0.1413	0.7353-1.0000
1.5 It meets the objectives of the project.	0.9167	91.67	0.4803	0.6152-0.9978
1.6 There is a logical sequence of content.	1.0000	100	0.1413	0.7353-1.0000
1.7 The information presented is scientifically correct.	0.8333	83.33	1.0000	0.5158-0.9791
1.8 The content is not repeated.	1.0000	100	0.1413	0.7353-1.0000
1.9 The content reflects the validated script.	1.0000	100	0.1413	0.7353-1.0000
**Organization**	0.9722	97.22	0.3446	0.2227-0.9567
2.1 The audio in the video is appropriate and helps with understanding the content.	1.0000	100	0.1413	0.7353-1.0000
2.2 The music is appropriate for the moment in which it is used.	1.0000	100	0.1413	0.7353-1.0000
2.3 The images in the video are appropriate for the content being covered.	0.9167	91.67	0.4803	0.6152-0.9978
2.4 The setting is appropriate.	0.9167	91.67	0.4803	0.6152-0.9978
2.5 The illustrations used are appropriate to the work content.	1.0000	100	0.1413	0.7353-1.0000
2.6 The image lighting and framing are appropriate.	1.0000	100	0.1413	0.7353-1.0000
**Writing style**	0.8888	88.88	0.4880	0.0942-0.9915
3.1 The participants in the video speak clearly.	0.8333	83.33	1.0000	0.5158-0.9791
3.2 The way they present themselves is appropriate.	1.0000	100	0.1413	0.7353-1.0000
3.3 The speeches are appropriate and reflect reality.	0.8333	83.33	1.0000	0.5158-0.9791
**TOTAL VALUE**	0.8991	89.91	0.0600	0.9107-0.9743

CVI - Content Validity Index; CI - Concordance Index;

* binomial test.

Concerningvalidity by media judges, seven media judges participated in this process. The majority were male (85.7%), were white (57.1%), and almost a third worked as photographers and video makers (28.6%). Regarding qualifications, specialization predominated (57.1%), and a total of 85.7% reported having previously published articles related to video production.

Table 2 shows the analysis of CVI, CI and binomial test regarding videovalidity by media judges. The total CVI of the video was 0.9, confirming video suitability and validity.

**Table 2:** Analysis of Content Validity Index, Concordance Index and binomial test regarding video validity by communication judges (n=7), Fortaleza, Ceará, Brazil, 2024

Instrument Questions	CVI	CI (%)	*p* value^*^	95% Confidence Interval
**Content**	0.9523	95.23%	0.396	0.2992-0.9251
1.1 The information/content is or is coherent.	1.000	100.00%	0.3577	0.5903-1.0000
1.2 The information/content is presented in a clear and understandable manner.	1.000	100.00%	0.3577	0.5903-1.0000
1.3 The way the content is presented in the video is inviting to the viewer.	0.7143	71.43%	0.6330	0.2904-0.9633
1.4 It can be circulated in the scientific community of the area.	1.000	100.00%	0.3577	0.5903-1.0000
1.5 It meets the project’sobjectives.	1.000	100.00%	0.3577	0.5903-1.0000
1.6 There is a logical sequence of content.	1.000	100.00%	0.3577	0.5903-1.0000
1.7 The information presented is scientifically correct.	1.000	100.00%	0.3577	0.5903-1.0000
1.8 The content is not repeated.	0.8571	85.71%	1.0000	0.4212-0.9963
1.9 The content reflects the validated script.	1.000	100.00%	0.3577	0.5903-1.0000
**Audio-visual**	0.9285	92.85%	0.0988	0.1181-0.8818
2.1 The audio in the video is appropriate and helps with understanding the content.	0.8571	85.71%	1.0000	0.4212-0.9963
2.2 The music is appropriate for the moment in which it is used.	1.000	100.00%	0.3577	0.5903-1.0000
2.3 The images in the video are appropriate for the content being worked on.	0.8571	85.71%	1.0000	0.4212-0.9963
2.4 The setting is appropriate.	1.000	100.00%	0.3577	0.5903-1.0000
2.5 The illustrations used are appropriate for the work content.	0.8571	85.71%	1.0000	0.4212-0.9963
2.6 The lighting and framing of the images are appropriate.	1.000	100.00%	0.3577	0.5903-1.0000
**Characters**	1.000	100.00%	1.0000	0.2924-1.0000
3.1 The participants in the video speak clearly.	1.000	100.00%	0.3577	0.5903-1.0000
3.2 The way they present themselves is appropriate.	1.000	100.00%	0.3577	0.5903-1.0000
3.3 The speeches are appropriate and reflect reality.	1.000	100.00%	0.3577	0.5903-1.0000
**TOTAL CVI**	0.9023	90.23%	0.3827	0.4651-0.9030

CVI - Content Validity Index; CI - Concordance Index;

* binomial test.

## DISCUSSION

The educational process in health becomes easier when a nurse uses instruments that aim to facilitate communication and understanding of individuals. Among other purposes, technologies can favor a better understanding of certain subjects and, more quickly, promote changes for individuals^(14)^. A proposal for valid educational technology to be used by nurses and healthcare professionals in the health education process of young black people about HIV testing is presented.

Educational technology, such as video, positively favors the teaching and learning process of young black people about rapid testing. This is due to the technology’s accessibility, interactivity, dynamism and attractiveness, facilitating the process of education and communication^(15)^.

In this research, the idea was to develop an educational video, produced based on the state of the art and the real need to involve two priority populations for HIV prevention. Thus, the video developed here presented important conceptual bases for understanding the topic. Language was appropriate for the young audience, and the video was a short film, lasting three minutes and 22 seconds, with the intention of making it available on various social media and allowing the young person to focus their attention on the video until it ended.

According to studies, the main technical characteristics of a video involve having a design similar to that of cartoons and being shorter than 15 minutes, since long videos can be tiring and can also lead to viewer distraction^(14,16)^.In the present study, we chose to include four animated characters, such as a nurse, responsible for nursing care and the health education process for young people, two young black people (Dandara, the main character, and Caio, the young woman’s friend) and a young white woman called Ruth.

The relevance of using educational videos for young black people on HIV prevention stands out. However, the development of this type of technology for this population, according to the analysis found through the scoping review of this study, showed only two international studies. These studies aimed, respectively, to test a cognitive-behavioral intervention to reduce the risk of HIV for heterosexually active African-American men and to reduce the sexual risk of HIV in urban women, predominantly African-American, through a series of 12 videos^(17,18)^.

In this regard, it is necessary and extremely important to develop and validate educational videos with experts on rapid testing as a way to prevent HIV in the young black population. In this research, a rigorous methodological approach was adopted to create and validate the educational video so that quality material could be developed. The judges who validated the video had direct experience with HIV and rapid testing, and half were black, and were therefore able to contribute relevant suggestions for improving the video.

In a study developed with the objective of mapping scientific production on educational strategies and the content covered in the education of people living with HIV, 17 studies were found. Most of studies sought to assess the impact and effectiveness of strategies and to develop or validate health education instruments and prevention activities. In this study, it was found that the educational strategies that stood out in relation to greater patient adherence to treatment were related to the development of systems, programs and multimedia resources, which supports educational video elaboration in this research^(19)^.

Another relevant aspect fortechnology construction was the use of clear, simple and accessible language to facilitate greater understanding by the target audience. According to literature, language in educational materials should avoid complex sentences, with a view to improving the understanding of a topic^(20)^.

In addition to the fluid language aspect, we sought to insert subtitles in the video, strengthening the learning process, as the subtitled video, which can contain subtitles of multi-semiotic texts, oral texts and sounds, favors the understanding of the topic^(21)^. Another important factor to highlight is the inclusion of LIBRAS in the video, providing accessibility to those who need it. In a study that sought to analyze the perceptions of 121 individuals with deafness in relation to the process of communicating with healthcare professionals in Primary Care in the state of Rio de Janeiro, it was found that the main barrier and difficulty was the absence of an intermediary to facilitate communication with professionals^(22)^.

In this context, the use of educational media and technologies becomes essential in the teaching and learning process for the deaf, as they produce different ways of understanding reality, learning, constructing and disseminating information^(23)^. It was from this perspective that the educational video for this study on rapid testing was developed.

### Study limitations

When carrying out the research, the limitations encountered were due to the difficulty in obtaining feedback from assessing judges, especially from social communication specialists.

### Contributions tonursing, health or public policy

The educational video developed and validated in this study by the target audience and health and social communication experts may contribute to health education actions developed within the scope of rapid testing for HIV, as well as other STIs and viral hepatitis involving the young and black population, and its dissemination may be accessed on online digital platforms with broad dissemination access. This will lead to advances in nursing practices, favoring the fight against the HIV epidemic, in addition to contributing to the reduction of HIV infection transmission in the young and black population.

## CONCLUSIONS

The educational video technology on rapid HIV testing for young black people is valid in terms of content and appearance, according to an assessment by experts in the field. The educational video produced presents a compilation of explanatory information for young black people and will be used in the health education process. The aim of this study is for nursing care using this technology to occupy a strategic position to favor the HIV epidemic control among young black people, while recognizing and understanding the social representations of this population regarding vulnerabilities and risk factors inherent to infection.

## Data Availability

The research data are available only upon request.
