# Supplementary material for: Construction and validity of an educational video on rapid HIV testing for young black people
Source: Rev Bras Enferm. 2025 Oct 3;78(Suppl 2):e20240409. doi: 10.1590/0034-7167-2024-0409 (PMC12500074; doi:10.1590/0034-7167-2024-0409)
Supplement: Translation

# Translation: Construção e validação de vídeo educativo sobre testagem rápida anti-HIV para jovens negros

**DOI:** 10.1590/0034-7167-2024-0409pt

**Authors:** Nikaelly Pinheiro Mota, Marli Teresinha Gimeniz Galvão, Nuno Damácio de Carvalho Félix, Renata Karina Reis, Ivana Cristina Vieira de Lima Maia, Rosely Leyliane dos Santos, Juliana Cunha Maia, Jéssica Karen de Oliveira Maia

**Affiliations:** I Universidade Federal do Ceará. Fortaleza, Ceará, Brasil; II Universidade Federal do Recôncavo da Bahia. Santo Antônio de Jesus, Bahia, Brasil; III Universidade de São Paulo. Ribeirão Preto, São Paulo, Brasil; IV Universidade Estadual do Ceará. Fortaleza, Ceará, Brasil; V Universidade Regional do Cariri. Crato, Ceará, Brasil

**Keywords:** Filme e Vídeo Educativo, Teste de HIV, Estudo de Validação, População Negra, Enfermagem.

## Abstract

**Objetivos:**

construir e validar vídeo educativo sobre testagem rápida anti-HIV para jovens negros.

**Métodos:**

pesquisa de produção tecnológica, desenvolvida pelas etapas: 1) revisão da literatura; 2) construção de roteiro; 3) validação de conteúdo; 4) construção do vídeo; e 5) validação deaparência. Calcularam-se os Índices de Validade de Conteúdo (IVC), Índice de Concordância (IC) e teste exato de binomial, considerando p>0,05 e a proporção de 0,80 de concordância.

**Resultados:**

o vídeo apresentou duração de três minutos e 22 segundos, abordando testagem rápida anti-HIV e *design* visual abrangendo animação, legendação e narração na Linguagem Brasileira de Sinais (LIBRAS). O IVC total do vídeo foi de 0,90 (valor de p=0,38; IC 95%: 0,46-0,90), confirmando a adequabilidade e validade.

**Conclusões:**

a tecnologia educacional, do tipo vídeo, sobre testagem rápida para HIV para jovens negros, é válida quanto ao conteúdo e à aparência, com potencial utilidade para ações de educação em saúde.

## INTRODUÇÃO

A vulnerabilidade social vivenciada pelas pessoas negras ocasiona desvantagens em saúde, em decorrência de alguns fatores como baixo nível de escolaridade e socioeconômico, número de pessoas sob dependência financeira e dificuldade de acesso aos serviços de saúde estarem mais prevalentes nesses indivíduos, elevando o risco para a exposição à infecção pelo HIV^(1)^.

Evidências epidemiológicas apontam que, entre 2007 e 2021, a taxa de infecção foi maior pelo vírus HIV em negros, sendo 51,7% em pretos e pardos^(2)^.Logo, tem-se o maior percentual de casos de infecção ao HIV entre jovens, tornando-se mais vulneráveis à infecção pelo vírus devido a situações diversas, como a precocidade da iniciação sexual, desejo de aceitação e inserção em grupos sociais, uso de álcool e outras drogas, questões de gênero, além de menor preocupação com a aquisição de infecções sexualmente transmissíveis (ISTs)^(3)^.

Isso posto, ressalta-se a relevância dos cuidados de enfermagem por ocuparem posição estratégica para favorecer o controle da epidemia de HIV entre a população jovem e negra, reconhecer e compreender as representações sociais dessa população sobre vulnerabilidades e os fatores de risco inerentes à infecção^(4)^.

De modo a permitir maior propagação de informações sobre a testagem rápida anti-HIV para os jovens negros, destaca-se a utilização de vídeos educativos que, por meio de elementos audiovisuais, como estímulo sonoro, visual e textual, tornam a tecnologia favorável para o processo de promoção da saúde. Além disso, o vídeo é uma tecnologia de fácil manuseio, estimulando a curiosidade, possibilitando a interação do participante e permitindo que o mesmo tenha a possibilidade de avançar, repetir, pausar conforme as demandas e ritmo de aprendizagem^(5)^.

As evidências científicas mapearam as tecnologias educativas desenvolvidas para a população negra, identificando tecnologias como cursos, mensagens, dramatização, aplicativos, panfletos, campanhas de mídia, campanhas de rádio, grupos de *Facebook^®^* e vídeos como intervenções junto à população negra, com impactos positivos^(6)^, mas com a lacuna no conhecimento sobre a construção de vídeos educativos válidos para a população negra envolvendo a testagem anti-HIV. Ressaltam-se o ineditismo e a originalidade deste estudo, haja vista não terem sido encontradas pesquisas nacionais publicadas para o público negro no que se refere ao desenvolvimento de tecnologia educativa que promova o conhecimento sobre testagem rápida anti-HIV.

Nesse sentido, a relevância científica e social desta pesquisa é fundamentada na necessidade de políticas públicas de saúde que visem proporcionar a ampliação de estratégias de cuidado, como a utilização de vídeos educativos e intervenções adequadas para o enfrentamento ao HIV nessa população. Com isso, o cuidado de enfermagem, aliado e reorientado a essas estratégias, torna-se de suma relevância para a disseminação de práticas direcionadas para o constructo da promoção e prevenção em saúde.

## OBJETIVOS

Construir e validar vídeo educativo sobre testagem rápida anti-HIV para jovens negros.

## MÉTODOS

### Aspectos éticos

A pesquisa obteve parecer favorável da Comissão de Ética em Pesquisa da Universidade Federal do Ceará, conforme estabelece a Resolução nº 466/12^(7)^.

### Desenho e período do estudo

Trata-se de pesquisa aplicada de produção tecnológica^(8)^, o qual envolveu a construção e validação de um vídeo educativo sobre testagem rápida anti-HIV para a população jovem negra.

As etapas realizadas no estudo envolveram: 1) Revisão de escopo para levantamento do corpus literário^(6)^; 2) Construção do *storyline* e roteiro do vídeo educativo; 3) Validação de conteúdo do roteiro do vídeo com juízes da saúde e população jovem negra (representantes do público-alvo); 4) Construção do vídeo educativo; e 5) Validação de aparência e características técnicas do vídeo educativo com juízes da saúde e da comunicação social. A pesquisa seguiu as diretrizes do instrumento SQUIRE 2.0 (Rede Equator) para nortear a metodologia, e foi realizada de dezembro de 2021 a outubro de 2023.

### População ou amostra; critérios de inclusão e exclusão

Para o desenvolvimento do vídeo, foi necessário o envolvimento de uma equipe multiprofissional especializada, na qual foi constituída por: autora da pesquisa para o desenvolvimento do *storyline*; roteirista, responsável pela confecção do roteiro; revisora de conteúdo, encarregada da revisão do roteiro e dos vídeos; produtora de áudio e vídeo, incumbida da produção do vídeo com legendação e locução dos personagens; e intérprete, para tradução do vídeo para a Língua Brasileira de Sinais (LIBRAS).

O processo de validação ocorreu em dois momentos: 1) Na validação do roteiro (com juízes da saúde *experts* na temática e por representantes do público-alvo sendo jovens negros de 18 a 29 anos); e 2) Na validação do vídeo (com juízes da saúde e da comunicação social).

Em ambos os momentos de validação, o processo ocorreu mediante amostragem não probabilística, de forma intencional, do tipo bola de neve^(8)^. A seleção dos juízes foi realizada mediante análise de currículos existentes na base de dados do Conselho Nacional de Desenvolvimento Científico (CNPq) e da experiência profissional nas respectivas áreas.Os critérios de inclusão dos juízes foram pessoas com experiência clínica e/ou pesquisas desenvolvidas na área em questão, especialista na estrutura conceitual envolvida e com conhecimento metodológico sobre a tecnologia a ser validada. No que se refere à quantidade necessária de juízes para o processo de validação, não há na literatura um consenso estabelecido^(9)^.

Os critérios de pontuação para seleção dos juízes da saúde consideraram a área de interesse relacionada ao HIV/AIDS/ISTs e testagens rápidas: ser doutor em área relacionada à temática de interesse (4 pontos); possuir tese na área de interesse (2 pontos); ser mestre em área relacionada à temática de interesse (3 pontos); possuir dissertação na área de interesse (2 pontos); possuir artigo publicado em periódico sobre a área de interesse (1 ponto); possuir prática profissional de, no mínimo, um ano na área de interesse (2 pontos). Requisitou-se a pontuação mínima de cinco pontos para ter sido convidado a participar do estudo^(10)^.

Na validação do roteiro, convidaram-se 25 juízes da área da saúde, mas apenas 13 aceitaram participar do estudo, e 15 jovens negros, obtendo-se dez respostas confirmatórias. Na validação do vídeo, convidaram-se os mesmos 25 juízes da área da saúde, mas apenas 12 deram continuidade nas avaliações, e quanto aos juízes da comunicação social, foram convidados 27, porém apenas sete participaram do estudo.

### Protocolo do estudo

Para a construção do vídeo, foram seguidas recomendações de referencial metodológico para elaboração de tecnologias audiovisuais, ocorrendo em três fases: pré-produção (revisão de escopo, criação do *storyline*, desenvolvimento do roteiro e validação do roteiro); produção; e pós-produção^(11)^.

Na pré-produção, desenvolveu-se revisão de escopo com objetivo de levantamento de *corpus* literário sobre as principais tecnologias desenvolvidas para a população negra no contexto do HIV, de modo a colaborar com o embasamento científico dos assuntos temáticos do vídeo. A revisão buscou responder à seguinte indagação: quais são as tecnologias educativas implementadas para prevenção do HIV em negros?

Esse processo de revisão ocorreu entre os meses de dezembro de 2021 e janeiro de 2022, sendo realizada busca de maneira ampla através do portal de periódicos da Coordenação de Aperfeiçoamento de Pessoal de Nível Superior (CAPES), acessando as bases de dados MEDLINE/PubMed, Embase, LILACS, CINAHL, Scopus e Cochrane^(6)^.

O conteúdo do *storyline* foi desenvolvido pela autora da pesquisa, havendo, subsequentemente, a criação do roteiro. O roteiro foi elaborado por meio da seleção de orientações disponibilizadas pelo Ministério da Saúde referentes à Mandala da Prevenção Combinada, especificamente sobre o item designado para as testagens rápidas. Associada às orientações do Ministério da Saúde, o conteúdo do roteiro apresentou ainda como base resultados obtidos por meio da revisão.

A construção do *storyline* ocorreu por meio de informações gerais sobre o que era imprescindível encontrar no vídeo. Não há na literatura um guia para nortear essa etapa de construção; assim, o seu desenvolvimento ficou a critério da pesquisadora.

Seguiu-se ainda na pré-produção com a validação do conteúdo pelos juízes selecionados. Os questionários adaptados utilizados para validação de conteúdo se caracterizaram por serem instrumentos de escala Likert, possuindo itens ou afirmativas a serem avaliadas pelos profissionais e público-alvo^(12)^.

Os questionários foram inseridos no *Google Forms* ^®^, associados aos seguintes documentos: carta-convite, Termo de Consentimento Livre e Esclarecido (TCLE) e Termo de Confidencialidade. O TCLE foi obtido de todos os juízes envolvidos no estudo de forma *online*, e o mesmo foi acessado por um *link* para solicitação do aceite da participação, de forma que o juiz recebesse em seu e-mail uma cópia do TCLE. Concedeu-se prazo de 15 dias para o retorno do material de coleta de dados. Após decorrido este prazo, a autora da pesquisa realizou a coleta dos dados.

No total, foram utilizados dois questionários para validação do roteiro, sendo um específico para os juízes da saúde, na qual avaliou objetivo, organização e relevância da tecnologia, e outro para o público-alvo, que buscou avaliar os objetivos, organização, e compreensão e estilo da escrita. Após a análise dos especialistas/público-alvo, acrescentaram-se sugestões, correções e/ou recomendações que julgaram importantes.

Na etapa de produção, ocorreu o desenvolvimento do vídeo educativo através de um estúdio de produção, gravação e edição de áudio e vídeo. O vídeo foi elaborado por meio de desenhos animados, apresentando a inclusão da LIBRAS.

Após a elaboração do vídeo, ocorreu novamente o processo de validação com os mesmos juízes da área da saúde, e houve ainda a participação de juízes da área técnica (profissionais de comunicação social com *expertise* na produção de vídeos). Do mesmo modo que na validação do roteiro, concedeu-se prazo de 15 dias para o retorno do material de coleta de dados, com abertura para acrescentarem sugestões, correções e/ou recomendações que fossem pertinentes.

A fase de pós-produção buscou o compartilhamento do vídeo nas principais plataformas digitais, como *YouTube^®^* (https://www.youtube.com/watch?v=-2RK6ufFNLI), *WhatsApp^®^* e *Instagram^®^*.

### Análise dos resultados e estatística

A análise foi realizada mediante os dados de cada avaliador, tanto dos juízes especialistas (saúde e comunicação social) quanto do público-alvo. Utilizou-se de estatística descritiva para análise das características dos juízes respondentes e das respostas dos questionários de avaliação do roteiro e do vídeo.

Houve cálculo do Índice de Validade de Conteúdo (IVC) de cada item do instrumento de avaliação do roteiro/vídeo, dos domínios do instrumento de avaliação do roteiro/vídeo e do IVC global (instrumento como um todo). Foi calculado o Índice de Concordância (IC), considerando-se validados os itens que obtiverem nível de concordância mínimo de 75% nas respostas positivas^(8)^. Para cálculo do IC, utilizou-se como parâmetro o número de vezes em que os avaliadores concordam em relação a cada uma das respostas, dividido pelo número total de avaliações, cujo resultado variasse entre 0% e 100%^(13)^.

Considerou-se válido o item cuja concordância entre os juízes foi ≥ 0,80^(8)^. Para verificar a concordância entre os juízes, utilizou-se o teste binomial, considerando-se haver concordância entre os juízes quando p>0,05. As análises foram realizadas no *software* Jamovi, versão 2.3.

## RESULTADOS

O vídeo apresentou duração de três minutos e 22 segundos, e foi intitulado “Testagem regular para HIV e outras IST e HV”. Encontra-se disponível em algumas mídias sociais (*Instagram^®^, WhatsApp^®^, Facebook^®^* e *YouTube^®^*). Houve sincronia dos componentes do vídeo (animação, legendação e narração em áudio e em libras), e a intérprete ficou exposta no canto inferior direito, conforme Figura 1.

**Figura 1:** Cenas do vídeo sobre testagem rápida anti-HIV para jovens negros, Fortaleza, Ceará, Brasil, 2024

O processo de validação ocorreu em dois momentos, sendo eles: a validação do roteiro por juízes da saúde e pelo público-alvo; e a segunda validação ocorreu após desenvolvimento do vídeo por meio dos mesmos juízes da saúde e por juízes da comunicação social com *expertise* na produção de vídeos.

Na validação do roteiro por juízes da saúde, participaram 13 juízes, sendo que a maioria era do gênero feminino (69,2%), tinha como profissão a enfermagem (92,3%) e exercia o cargo de enfermeiro assistencial na instituição (69,2%). Quanto à titulação, mais de um terço referiu ser mestre (46,1%).

O IVC total do instrumento de avaliação foi 0,8, contudo o valor do p foi igual a p=0,02, sinalizando que não houve concordância de todos os juízes em relação à adequabilidade do roteiro do vídeo.

Um dos juízes abordou que o roteiro está adequado na perspectiva de estimular os jovens a fazerem os testes. Contudo, sugeriu que poderia acrescentar na parte do aconselhamento informações sobre o uso do preservativo em toda relação sexual (oral, vaginal ou anal) e sobre as outras formas de contaminação pelo HIV. Outra sugestão foi que poderia trazer sobre formas de adquirir o vírus e como prevenir (preservativo interno ou externo em todas as relações sexuais - oral, vaginal ou anal). Outro juiz enfatizou que é importante melhorar o contexto das cenas, incluindo especificidades do público trabalhado.

Salienta-se que a proposta do vídeo do presente estudo vislumbra abordar a temática “testagem rápida”, não o uso de preservativos. Assim, alguns apontamentos foram de suma relevância para mudanças e ajustes necessários na segunda versão do roteiro, como adicionar mais especificidade nas cenas quanto às características étnicas e raciais do público-alvo.

A segunda validação do roteiro ocorreu por meio do público-alvo (jovens negros). Em relação à caracterização do público-alvo (n=10) que participou da validação do roteiro, predominou o gênero feminino (70%), da raça parda (70%), com ensino superior completo (40%) ou incompleto (40%), sendo que 40% eram técnicos de enfermagem e 30% referiram ter como profissão a enfermagem. Houve concordância unânime (100%) pelo público-alvo em relação ao roteiro. O IVC total das respostas do instrumento de avaliação do vídeo pelo público-alvo foi de 0,9 (p=0,4), demonstrando a concordância do público-alvo em relação à adequabilidade do roteiro do vídeo.

No que se refere à validação do vídeo educativo, os resultados do IVC, IC e teste binomial na validação dos juízes, ocorreu avaliação nas seguintes categorias: conteúdo; audiovisual; e personagens.

Os juízes da saúde eram predominantemente do gênero feminino (75%), sendo metade (50%) da raça/cor parda e metade da raça/cor branca (50%). A maior parte dos respondentes respondeu que tinha a enfermagem como profissão (83,3%) e atuava como enfermeiro assistencial (66,7%). Quanto à titulação, 41,7% tinham mestrado, e 41,7%, doutorado. Nenhum dos participantes referiu ter publicação prévia de artigos sobre saúde da população negra.

Na Tabela 1, observam-se o IVC, IC e teste binomial em relação à validação do vídeo pelos juízes da saúde. De acordo com o observado, todos os itens individuais da avaliação do vídeo tiveram IVC superior a 0,8 e IC maior que 80%. Esse foi confirmado ao se realizar o teste binomial, que demonstrou concordância dos juízes da saúde em relação a todos os itens do instrumento de avaliação do vídeo, sendo todos os valores superiores a 0,05. O IVC total das respostas do instrumento de avaliação do vídeo foi 0,8 (p=0,06), demonstrando a concordância dos juízes da saúde acerca da adequação do vídeo.

**Tabela 1:** Índice de Validade de Conteúdo, Índice de Concordância e teste binomial em relação à validação do vídeo pelos juízes da saúde (n=12), Fortaleza, Ceará, Brasil, 2024

Perguntas do instrumento	IVC	IC (%)	Valor de *p* ^*^	Intervalo de Confiança de 95%
**Objetivo**	0,9537	95,37	0,0856	0,2120-0,8630
1.1 As informações/conteúdo são ou estão coerentes.	1,0000	100,00	0,1413	0,7353-1,0000
1.2 As informações/conteúdo são apresentadas de forma clara e compreensível.	0,9167	91,67	0,4803	0,6152-0,9978
1.3 A forma de apresentação do conteúdo no vídeo é convidativa para quem assiste.	0,9167	91,67	0,4803	0,6152-0,9978
1.4 Pode circular no meio científico da área.	1,0000	100	0,1413	0,7353-1,0000
1.5 Atende aos objetivos do projeto.	0,9167	91,67	0,4803	0,6152-0,9978
1.6 Há uma sequência lógica do conteúdo.	1,0000	100	0,1413	0,7353-1,0000
1.7 As informações apresentadas estão cientificamente corretas.	0,8333	83,33	1,0000	0,5158-0,9791
1.8 O conteúdo não se repete.	1,0000	100	0,1413	0,7353-1,0000
1.9 O conteúdo reflete o roteiro validado.	1,0000	100	0,1413	0,7353-1,0000
**Organização**	0,9722	97,22	0,3446	0,2227-0,9567
2.1 O áudio do vídeo está adequado e colabora na compreensão do conteúdo.	1,0000	100	0,1413	0,7353-1,0000
2.2 As músicas estão adequadas ao momento em que são utilizadas.	1,0000	100	0,1413	0,7353-1,0000
2.3 As imagens que compõem o vídeo são adequadas ao conteúdo trabalhado.	0,9167	91,67	0,4803	0,6152-0,9978
2.4 O cenário está apropriado.	0,9167	91,67	0,4803	0,6152-0,9978
2.5 As ilustrações utilizadas estão adequadas ao conteúdo do trabalho.	1,0000	100	0,1413	0,7353-1,0000
2.6 A iluminação e enquadramento das imagens estão adequados.	1,0000	100	0,1413	0,7353-1,0000
**Estilo de escrita**	0,8888	88,88	0,4880	0,0942-0,9915
3.1 Os participantes do vídeo falam com clareza.	0,8333	83,33	1,0000	0,5158-0,9791
3.2 A forma como que se apresentam está adequada.	1,0000	100	0,1413	0,7353-1,0000
3.3 As falas estão adequadas e refletem a realidade.	0,8333	83,33	1,0000	0,5158-0,9791
**VALOR TOTAL**	0,8991	89,91	0,0600	0,9107-0,9743

IVC - Índice de Validade de Conteúdo; IC - Índice de Concordância;

* teste binomial.

Quanto à validação pelos juízes da comunicação social, sete juízes da comunicação participaram nesse processo. A maioria era do gênero masculino (85,7%), tinha raça/cor branca (57,1%), e quase um terço trabalhava como fotógrafo e *videomaker* (28,6%). Quanto à titulação, predominou a especialização (57,1%), e um total de 85,7% referiu ter publicação prévia de artigos relacionados à produção de vídeo.

A Tabela 2 traz a análise do IVC, IC e teste binomial acerca da validação do vídeo pelos juízes da comunicação. O IVC total do vídeo foi de 0,9, confirmando a adequabilidade e validade do vídeo.

**Tabela 2:** Análise do Índice de Validade de Conteúdo, Índice de Concordância e teste binomial acerca da validação do vídeo pelos juízes da comunicação (n=7), Fortaleza, Ceará, Brasil, 2024

Perguntas do instrumento	IVC	IC	Valor de *p* ^*^	Intervalo de Confiança de 95%
**Conteúdo**	0,9523	95,23%	0.396	0,2992-0,9251
1.1 As informações/conteúdo são ou estão coerentes.	1,000	100,00%	0,3577	0,5903-1,0000
1.2 As informações/conteúdo são apresentadas de forma clara e compreensível.	1,000	100,00%	0,3577	0,5903-1,0000
1.3 A forma de apresentação do conteúdo no vídeo é convidativa para quem assiste.	0,7143	71,43%	0,6330	0,2904-0,9633
1.4 Pode circular no meio científico da área.	1,000	100,00%	0,3577	0,5903-1,0000
1.5 Atende aos objetivos do projeto.	1,000	100,00%	0,3577	0,5903-1,0000
1.6 Há uma sequência lógica do conteúdo.	1,000	100,00%	0,3577	0,5903-1,0000
1.7 As informações apresentadas estão cientificamente corretas.	1,000	100,00%	0,3577	0,5903-1,0000
1.8 O conteúdo não se repete.	0,8571	85,71%	1,0000	0,4212-0,9963
1.9 O conteúdo reflete o roteiro validado.	1,000	100,00%	0,3577	0,5903-1,0000
**Audiovisual**	0,9285	92,85%	0,0988	0,1181-0,8818
2.1 O áudio do vídeo está adequado e colabora na compreensão do conteúdo.	0,8571	85,71%	1,0000	0,4212-0,9963
2.2 As músicas estão adequadas ao momento em que são utilizadas.	1,000	100,00%	0,3577	0,5903-1,0000
2.3 As imagens que compõem o vídeo são adequadas ao conteúdo trabalhado.	0,8571	85,71%	1,0000	0,4212-0,9963
2.4 O cenário está apropriado.	1,000	100,00%	0,3577	0,5903-1,0000
2.5 As ilustrações utilizadas estão adequadas ao conteúdo do trabalho.	0,8571	85,71%	1,0000	0,4212-0,9963
2.6 A iluminação e enquadramento das imagens estão adequados.	1,000	100,00%	0,3577	0,5903-1,0000
**Personagens**	1,000	100,00%	1,0000	0,2924-1,0000
3.1 Os participantes do vídeo falam com clareza.	1,000	100,00%	0,3577	0,5903-1,0000
3.2 A forma como que se apresentam está adequada.	1,000	100,00%	0,3577	0,5903-1,0000
3.3 As falas estão adequadas e refletem a realidade.	1,000	100,00%	0,3577	0,5903-1,0000
**IVC TOTAL**	0,9023	90,23%	0,3827	0,4651-0,9030

IVC - Índice de Validade de Conteúdo; IC - Índice de Concordância;

* teste binomial.

## DISCUSSÃO

O processo educacional em saúde se torna facilitado quando o enfermeiro utiliza instrumentos que tenham por finalidade facilitar a comunicação e o entendimento dos indivíduos. Entre outras finalidades, as tecnologias podem favorecer o melhor entendimento sobre determinados assuntos e, de maneira mais breve, promover mudanças para os indivíduos^(14)^. Apresenta-se uma proposta de tecnologia educacional válida a ser utilizada por enfermeiros e profissionais de saúde no processo de educação em saúde de jovens negros sobre a testagem anti-HIV.

A tecnologia educacional, do tipo vídeo, favorece, de forma positiva, o processo de ensino aprendizagem de jovens negros sobre testagem rápida. Isso se deve à acessibilidade, interatividade, dinamicidade e atratividade da tecnologia, facilitando o processo de educação e comunicação^(15)^.

Nesta pesquisa, idealizou-se desenvolver um vídeo educativo, produzido a partir do estado da arte e da real necessidade de envolver duas populações prioritárias para prevenção do HIV. Assim, o vídeo ora desenvolvido apresentou bases conceituais importantes para o entendimento da temática. Houve adequabilidade da linguagem para o público jovem, e o tempo de duração do vídeo foi de curta metragem, com duração de três minutos e 22 segundos, tendo a pretensão de disponibilização em diversas mídias sociais e que permitisse que o jovem concentrasse sua atenção no vídeo até seu término.

Conforme estudos, as principais características técnicas de um vídeo envolvem possuir *design* similar à de desenhos animados e a duração ser menor que 15 minutos, visto que vídeos extensos podem ser cansativos, bem como podem induzir à dispersão do espectador^(14,16)^. No presente estudo, optou-se pela inclusão de quatro personagens animados, tais como um enfermeiro, responsável pelo cuidado de enfermagem e processo de educação em saúde aos jovens, dois jovens negros (Dandara, sendo a personagem principal, e Caio, amigo da jovem) e uma jovem branca chamada Ruth.

Ressalta-se a relevância da utilização de vídeos educativos para jovens negros sobre prevenção do HIV. Entretanto, o desenvolvimento desse tipo de tecnologia para esta população, conforme análise encontrada por meio da revisão de escopo deste estudo, evidenciou apenas dois estudos internacionais. Nestes estudos, objetivou-se, respectivamente, testar uma intervenção cognitivo-comportamental de redução do risco de HIV para homens afro-americanos heterossexualmente ativos e reduzir o risco sexual de HIV em mulheres urbanas, predominantemente afro-americanas, por meio de uma série de 12 vídeos^(17,18)^.

Nesse sentido, faz-se necessário e caracteriza-se como de suma relevância o processo de desenvolvimento e validação com especialistas de vídeos educativos voltados para a temática da testagem rápida como forma de prevenção do HIV na população negra e jovem. Nesta pesquisa, adotou-se um percurso metodológico rigoroso, utilizado na construção e validação do vídeo educativo, para que houvesse o desenvolvimento de um material de qualidade. Os juízes que realizaram a validação do vídeo tinham experiência direta com HIV e testagem rápida, e a metade era negra, podendo, assim, contribuir com sugestões relevantes para o aprimoramento do vídeo.

Em estudo desenvolvido com objetivo de mapear a produção científica sobre as estratégias educativas e os conteúdos abordados na educação de pessoas vivendo com HIV, foram encontrados 17 estudos. A maioria dos estudos buscou avaliar o impacto e a eficácia das estratégias e desenvolver ou validar instrumentos de educação em saúde e atividades de prevenção. Nesse estudo, constatou-se que as estratégias educativas que se destacaram em relação à maior adesão dos pacientes ao tratamento estavam relacionadas ao desenvolvimento de sistemas, programas e recursos multimídia, o que corrobora a elaboração do vídeo educativo nesta pesquisa^(19)^.

Outro aspecto relevante para construção da tecnologia foi a utilização de linguagem clara, simples e acessível para facilitar o maior entendimento do público-alvo. De acordo com a literatura, a linguagem nos materiais educativos deve evitar frases complexas, com vistas a melhorar a compreensão de uma temática^(20)^.

Além do aspecto da linguagem de forma fluida, buscou-se inserir legendas no vídeo, fortalecendo o processo de aprendizagem, pois o vídeo legendado, que pode conter legendas de textos multissemióticos, de textos orais e de sons, favorece a compreensão da temática^(21)^. Outro fator importante a destacar é a inclusão da LIBRAS no vídeo, proporcionando acessibilidade aos que necessitam. Em estudo que buscou analisar as percepções de 121 indivíduos com surdez em relação ao processo de comunicação com profissionais de saúde da Atenção Básica do estado do Rio de Janeiro, constatou-se que a principal barreira e dificuldade foi a ausência de um intermediador para facilitar a comunicação com os profissionais^(22)^.

Nesse contexto, torna-se indispensável no processo de ensino e aprendizagem para surdos a utilização de mídias e tecnologias educacionais, pois produzem diferentes formas de compreender a realidade, de aprender, construir e disseminar informações^(23)^. Foi nessa perspectiva que o vídeo educativo deste estudo sobre testagem rápida foi desenvolvido.

### Limitações do estudo

Na execução da pesquisa, as limitações encontradas foram decorrentes da dificuldade de obter retorno de respostas por parte dos juízes avaliadores, principalmente por parte dos especialistas na área da comunicação social.

### Contribuições para as áreas da enfermagem, saúde ou políticas públicas

O vídeo educativo desenvolvido e validado neste estudo pelo público-alvo e especialistas da saúde e comunicação social poderá contribuir com as ações de educação em saúde desenvolvidas no âmbito das testagens rápidas para HIV, além de outras ISTs e hepatites virais envolvendo a população jovem e negra, e sua divulgação poderá ser acessada em plataformas digitais *online* com amplo acesso de divulgação. Com isso, haverá avanços nas práticas pelo enfermeiro, favorecendo o enfrentamento da epidemia do HIV, além de colaborar para a redução da transmissão da infecção pelo HIV na população jovem e negra.

## CONCLUSÕES

A tecnologia educacional, do tipo vídeo, sobre testagem rápida para HIV para população jovem negra, é válida quanto ao conteúdo e à aparência, em conformidade com avaliação por especialistas da área. O vídeo educativo produzido apresenta um compilado de informações de caráter explicativo para a população jovem negra e servirá para o processo de educação em saúde. Almeja-se, com este estudo, que os cuidados de enfermagem a partir dessa tecnologia ocupem posição estratégica para favorecer o controle da epidemia de HIV entre a população jovem e negra, ao passo de reconhecer e compreender as representações sociais dessa população sobre vulnerabilidades e os fatores de risco inerentes à infecção.

## Data Availability

Os dados de pesquisa só estão disponíveis mediante solicitação.
